# The Effect of the Stress-Signalling Mediator Triacontanol on Biochemical and Physiological Modifications in *Dracocephalum forrestii* Culture

**DOI:** 10.3390/ijms232315147

**Published:** 2022-12-02

**Authors:** Izabela Weremczuk-Jeżyna, Katarzyna Hnatuszko-Konka, Liwia Lebelt, Dorota G. Piotrowska, Izabela Grzegorczyk-Karolak

**Affiliations:** 1 Department of Biology and Pharmaceutical Botany, Faculty of Pharmacy, Medical University of Lodz, Muszyńskiego 1, 90-151 Lodz, Poland; 2 Department of Molecular Biotechnology and Genetics, Faculty of Biology and Environmental Protection, University of Lodz, Banacha 12/16, 90-237 Lodz, Poland; 3 Bioorganic Chemistry Laboratory, Faculty of Pharmacy, Medical University of Lodz, Muszyńskiego 1, 90-151 Lodz, Poland

**Keywords:** biologically active compounds, *Dracocephalum forestii*, in vitro culture, phytohormones, plant secondary metabolites, triacontanol, rosmarinic acid

## Abstract

Triacontanol (TRIA) has been reported to influence signal transduction in the crosstalk triggered by various stress factors. As a signal player, it is also known to affect many physiological processes, including enhancing the biosynthesis of secondary metabolites. Such knowledge can be used to direct or boost the production of bioactive secondary compounds without stress induction. Therefore, the aim of this study is to evaluate the use of TRIA as a factor stimulating the growth and production of bioactive compounds in the shoot culture of *Dracocephalum forrestii*. TRIA was applied at three concentrations (2.5, 5, and 10 µM), alone or in combination with phytohormones (6-benzylaminopurine and indole-3-acetic acid). After five weeks, growth and physiochemical parameters (chlorophyll content, antioxidant enzyme activity, and phenolic acid level) were determined. The results indicate that TRIA application significantly increased shoot dry weight, chlorophyll content, antioxidant enzyme activities (superoxide dismutase, peroxidase, and catalase), and total polyphenol level; it also influenced the multiplication ratio in combination with growth regulators. The greatest antioxidant enzyme activity was observed for 5 µM TRIA in hormone-free medium, while the most significant secondary metabolite production was obtained for phytohormone-containing medium supplemented with 10 µM TRIA: total phenolic acid content (19.4 mg/g dry weight) was twice that of the control. Hence, the TRIA application appears to be a valuable biotechnology technique for modifying plant metabolite production.

## 1. Introduction

Plant cell culture is a common alternative to conventional propagation for producing biomass and biologically-important secondary metabolites. The growth and productivity of in vitro cultures can be regulated, inter alia, by adding various exogenous compounds, particularly plant growth regulators (PGRs) such as cytokinins, auxins or gibberellins. These substances can modulate morphological, biochemical, photosynthetic, and developmental processes [1]. PGRs are effective at low concentrations and affect cell division, tissue regeneration, plant organ formation, and plant reproduction. Phytohormones and their synthetic equivalents also regulate the pathways of plant secondary metabolites by influencing the production and expression of appropriate enzymes [2]. However, similar effects can be obtained in vitro by some biostimulants [3]. These natural or synthetic substances also influence plant growth and modify or regulate different physiological processes when given in small concentrations.

A natural plant biostimulant is triacontanol (TRIA) [4]. It is an aliphatic thirty carbon alcohol used as a growth promotor and is commonly applied to horticultural and agricultural crops [5]. It can be obtained from epicuticular waxes in several plant species, such as *Medicago sativa*, *Vaccinium ashei* or *Oryza sativa* [6,7,8]. TRIA application was found to significantly increase fresh and dry weight, leaf area, and plant weight [9,10]. It is believed to act by influencing various plant biochemical and physiological processes; for example, it has been found to regulate photosynthesis by enhancing the activity of rbcS genes and ribulose-1,5 bisphosphate carboxylase oxygenase (RuBisCO) [11]. Many of these processes occur in response to diverse stress factors, and TRIA influences signal transduction in their crosstalk. Namely, TRIA can enhance the activity of antioxidant enzymes, i.e., peroxidase (POD), glutathione reductase (GR), superoxide dismutase (SOD), and catalase (CAT) occurring in response to stress factors such as heavy metals, salts, water or temperature [12]. Some studies indicate that TRIA may also influence the content of important secondary metabolites in many plant species [9,13]; it may play a role in the interactions occurring between environmental factors and influence the status of plant secondary metabolites.

Due to the wide range of properties, attempts have been made to use TRIA in in vitro plant cultures [14,15]. Several works reported that the addition of TRIA to a growth medium, alone or in combination with other plant growth regulators, affected propagation and shoot growth. Cultures treated with TRIA typically demonstrated a higher number of shoots per explant and higher biomass compared to controls, and the addition of TRIA to the growth medium increased the level of some bioactive metabolites. For example, in vitro shoots of *Salvia officinalis* and *Thymus mastichiana* accumulated higher levels of phenolic compounds and essential oil constituents, respectively, compared to controls [14,15]. It proves that the impact of biotic stress factors on plant-specialized metabolites and general plant conditions can be demonstrated indirectly through the signal molecule application. The analysis of stressor-induced signalling pathways allows for the selection of significant molecular players involved (here, e.g., TRIA) that subsequently can be used as a tool for gaining desired modifications in plant physiology and production. Hence, without stress induction, different changes in plant growth rate and in in vitro production of secondary metabolites can be achieved.

Since TRIA is known to influence many physiological processes, and to enhance the biosynthesis of secondary metabolites, the aim of this study is to evaluate the use of TRIA as a factor stimulating the growth and production of bioactive compounds in *Dracocephalum forrestii*—the endemic Chinese medicinal plant. This species grows in mountainous areas, over 2000 m above sea level, and its aboveground parts are traditionally used in folk medicine as an astringent, diuretic, and antipyretic factor [16,17]. *D. forrestii* shoot culture is a valuable source of polyphenolic compounds, particularly phenolic acids (e.g., rosmarinic acid, caffeic acid, chlorogenic acid, salvianolic acid B) [18]. The presence of these compounds in plant material determines its antioxidant, antimicrobial, and antitumor properties [19].

The present study evaluates the effect of different concentrations of TRIA on the proliferation and biomass of *D. forrestii* shoots, applied alone or in combination with other phytohormones: 0.5 mg/L 6-benzylaminopurine (BAP) and 0.2 mg/L indole-3-acetic acid (IAA). It examines the influence of TRIA on the physiological processes of plant cells, with regard to chlorophyll production and the activity of antioxidant enzymes. The aim of the study was also to determine the optimal content of TRIA for the production of phenolic acids in *D. forrestii* in vitro culture.

## 2. Results and Discussion

### 2.1. Effects of TRIA on Culture Growth

**Figure 1:**
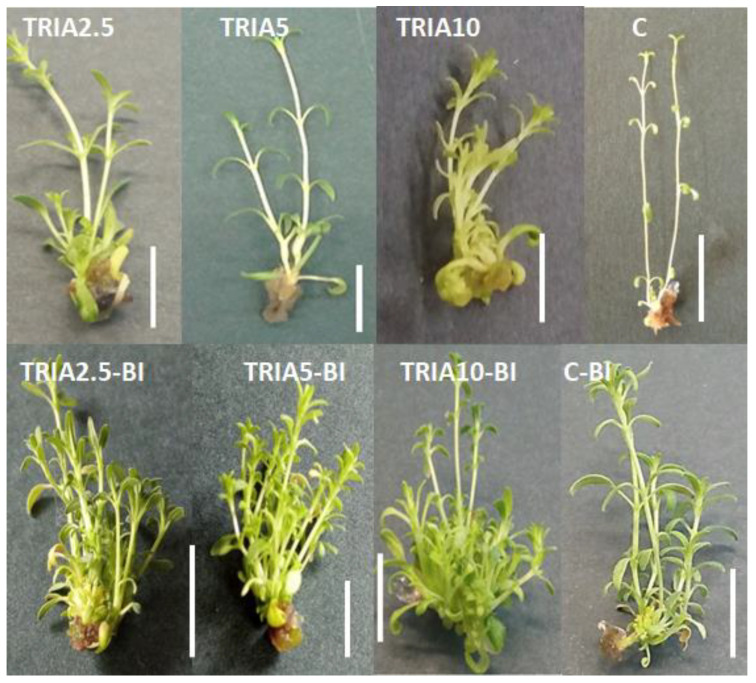
*D. forrestii* shoots growing five weeks on MS medium containing: TRIA (µg/L) or TRIA (µg/L) and 0.5 mg/L BAP and 0.2 mg/L IAA (TRIA-BI), C-medium without growth regulators, C-BI- medium with 0.5 mg/L BAP and 0.2 mg/L IAA. Bar 1 cm.

The nodal segments of *D. forrestii* shoots were cultivated on MS (Murashige and Skoog’s medium) agar medium containing 2.5, 5, or 10 µg/L triacontanol, with or without a growth hormone mix (0.5 mg/L BAP and 0.2 mg/L IAA). Shoots cultivated in simple MS medium and on MS medium containing the auxin-cytokinin mix (0.5 mg/L BAP and 0.2 mg/L IAA) were treated as controls. No difference in the percentage of response was found between plants grown on the media used for the shoot cultivation; for all media variants, the percentage of explants forming shoots ranged from 85 to 100% (Figure 1).

Media supplemented with growth regulators stimulated callus formation on *Dracocephalum* shoots; 5% explants grown on control C-BI medium formed callus tissue. The addition of TRIA to the phytohormone-containing medium increased the frequency of callus formation to 40% (Figure 1). The obtained callus tissue was undifferentiated and showed no tendency to regenerate organs. Previous studies indicate that triacontanol can promote callus formation: Hanganter and Ries [20] observed induction of undifferentiated *Nicotiana tabacum* callus at 10 µg /disc TRIA, and Abhirami et al. [21] noted callus induction on *A. gangetica* leaves cultivated on media with 1–20 µg/L TRIA. However, no callus formed on *D. forrestii* explants in the presence of TRIA alone, i.e., without phytohormone in the medium.

**Figure 2:**
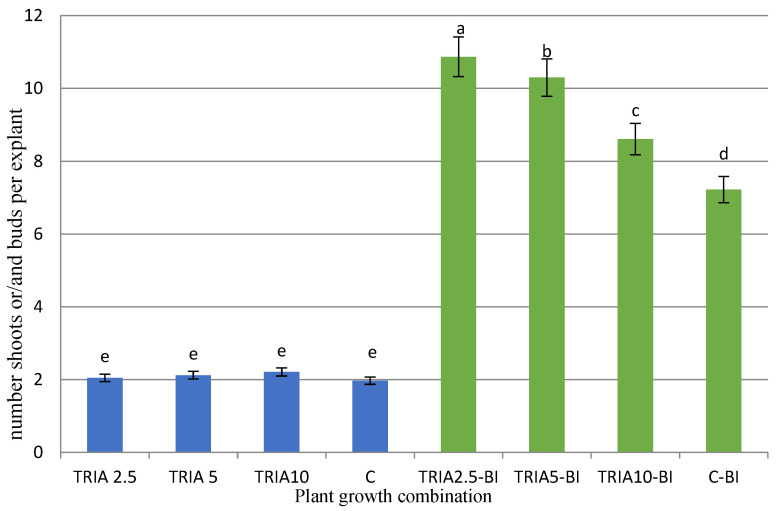
Effect of triacontanol on the propagation of *D. forrestii* shoots cultured five weeks on MS medium containing TRIA (µg/L) or TRIA (µg/L) and 0.5 mg/L BAP and 0.2 mg/L IAA (TRIA-BI): C-medium without growth regulators, C-BI- medium with 0.5 mg/L BAP and 0.2 mg/L IAA. The values represented means ± SE of three independent experimental replicates. The means marked with various letters for the same parameter were different at *p* < 0.05 according to Tukey’s test.

The addition of TRIA to the MS medium, regardless of its concentration, did not influence the proliferation of *D. forrestii* shoots. For all TRIA treatments without the IAA/BAP mix and for the control C, the proliferation ratio was about two shoots per explant (Figure 2). However, when triacontanol was added to the BAP-containing medium, it enhanced the shoot regeneration effect. The highest shoot proliferation ratio was found on medium with 2.5 µg/L TRIA, 0.5 mg/L BAP and 0.2 mg/L IAA, i.e., 10 new shoot/buds per explant. This value was about 50% higher compared to that obtained for shoots grown on MS medium without TRIA (C-BI) and five times higher than that for the culture on MS medium (C) (Figure 2). Increasing the TRIA concentration in media with BAP and IAA gradually reduced the proliferation ratio; however, the proliferation ratio remained greater than the control C-BI value, even in the medium with the highest TRIA level (10 µg/L) (Figure 2).

TRIA was found to promote in vitro multiplication for other medicinal plants in the *Lamiaceae*, such as *Melissa officinalis*, *Salvia officinalis*, and *Thymus masthiana* [14,15,22]. TRIA used at concentrations between 2–10 µg/L increased the proliferation of the above species by up to 50%. Similarly, in the case of *M. officinalis*, the lowest concentration (2 µg/L) was found to yield the best results [22]. In contrast, for the other two species, the highest multiplication ratio was observed for the higher TRIA concentration [14,15].

**Figure 3:**
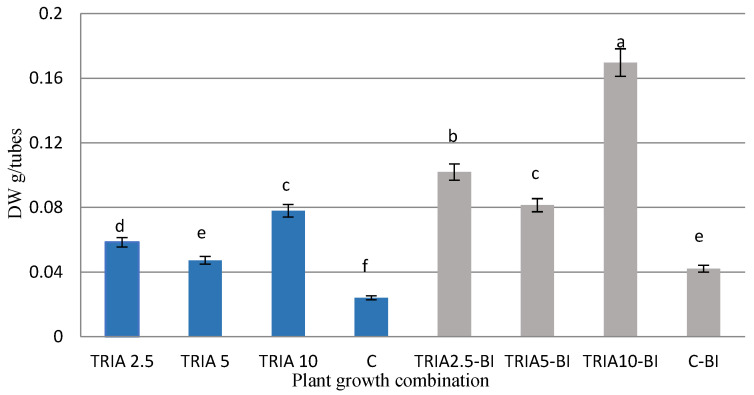
Effect of TRIA on dry mass (DW) of *D. forrestii* shoots, growing five weeks on MS medium containing TRIA (µg/L) or TRIA (µg/L) and 0.5 mg/L BAP and 0.2 mg/L IAA (TRIA-BI): C-medium without growth regulators, C-BI- medium with 0.5 mg/L BAP and 0.2 mg/L IAA. The values represented means ± SE of three independent experimental replicates. The means marked with various letters for the same parameter were different at *p* < 0.05 according to a Tukey’s test.

In the present study, TRIA was found to influence biomass growth in *D. forrestii*. Supplementation significantly increased the dry weight of the culture in comparison with the control culture (C) at all used concentrations (Figure 3). Regardless of the proliferation ratio, the *D. forrestii* shoots growing on the TRIA-supplemented media were thicker and have larger leaves than those growing on the control media. The highest dry weight was obtained when shoots were cultivated on medium with 10 µg/L TRIA (0.078 g/tubes). In this case, the dry weight increased 40-fold within five weeks, i.e., four times higher than for the control. Also, supplementation of the MS medium with the auxin-cytokinin complex increased in culture dry weight (C vs. C-BI), and TRIA additionally intensified the action of growth regulators. Again, the highest TRIA concentration used (10 µg/L) proved to be the most effective (Figure 3). The dry biomass (0.17 g/tubes) was found to be four times higher than for the control (C-BI), i.e., an 80- fold increase in dry weight within five weeks.

It has previously been noted that 10 µg/L TRIA combined with BAP and IAA most effectively stimulated the growth of *S. officinalis* shoots [14]. The presence of TRIA in the medium also stimulated the biomass of in vitro cultivated *T. masthiana* and *Capsicum frutescens* shoots [15,23].

Earlier reports suggest that the observed increase in biomass and multiplication ratio of plants treated with TRIA could result from the formation of the second messenger 9-ß-L (+) adenosine, with a similar structure to purine cytokinins [5,6]. The 9-ß-L (+) adenosine influences the physio-biochemical processes related to plant growth. It leads to the formation and proliferation of cells and induces activities of enzymes relating to carbohydrate and protein metabolism in plants, and the increase in plant dry weight may result from an increase in the levels of reducing sugars, soluble proteins, and free amino acids in the plant [6]. Additionally, TRIA promotes growth by inducing other substances stimulating plant development, such as gibberellic acid, IAA, glucosamine, melatonin, and serotonin [12].

### 2.2. Determination of Chlorophyll Content

**Figure 4:**
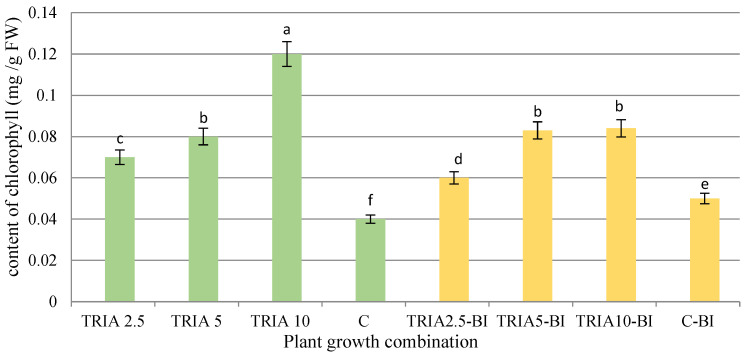
Effect of triacontanol on chlorophyll content in shoots of *D. forrestii* cultured for five weeks on MS medium containing TRIA (µg/L) or TRIA (µg/L) and 0.5 mg/L BAP and 0.2 mg/L IAA (TRIA-BI), C-medium without growth regulators, C-BI- medium with 0.5 mg/BAP and 0.2 mg/L IAA. The values represented means ± SE of three independent experimental replicates. The means marked with various letters for the same parameter were different at *p* < 0.05 according to a Tukey’s test.

Chlorophyll content was significantly enhanced in the *D. forrestii* shoots after the addition of TRIA into MS medium; shoots treated with 10 µg/L demonstrated three-fold higher levels compared to control shoots (0.12 mg/g fresh weight (FW)) (Figure 4). Moreover, shoots grown on media with TRIA in combination with phytohormones demonstrated higher amounts of chlorophyll compared to control (C-BI) (Figure 4). Also, in this case, a higher chlorophyll concentration was observed at higher TRIA concentration, reaching a maximum at TRIA 10 µg/L (about 0.085 mg/g FW). However, the presence of growth regulators in the growth media significantly reduced the stimulating effect of TRIA on chlorophyll production.

Similarly, Tantos et al. [22] found the amount of photosynthetic pigments to increase with increasing TRIA concentration in *M. officinalis* shoots in vitro. The highest amount of chlorophyll was observed in shoots grown on medium with 10 µg/L TRIA, i.e., 176% of the amount observed in the control. Previous studies attribute the increase of photosynthetic pigments in plants treated with TRIA to the crucial role it plays in protecting protein-pigment complexes and up-regulating the *rbcS* genes related to chlorophyll biosynthesis [11,24].

### 2.3. Genetic Stability

Several molecular methods, such as random amplified polymorphic DNA (RAPD), restriction fragment length polymorphism (RFLP) or inter simple sequence repeats (ISSR), have been used to analyze the genetic stability within tissue culture-regenerated shoots. In this case, ISSR amplification was chosen due to its simplicity, cost-effectiveness, and usefulness in the analysis of non-sequenced genomes. Hence, the genetic variation of both the control cultures and investigated regenerants were analyzed by the analysis of the band reproducibly. The amplification yielded 264 scorable bands, generated with six randomly-selected single microsatellite primers (Table S1).

**Figure 5:**
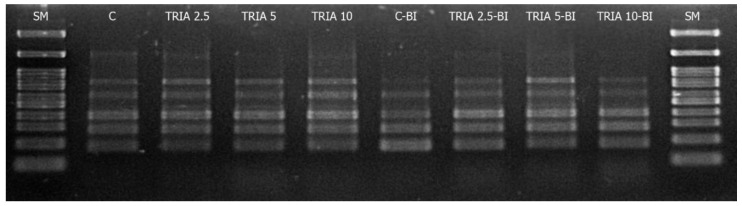
ISSR profile of shoot culture of *D. forrestii*—results for primer 840; lanes: SM—Fast gene ruler 100 bp, MS medium containing: TRIA (µg/L) or TRIA (µg/L) and 0.5 mg/L BAP and 0.2 mg/L IAA (TRIA-BI), C-medium without growth regulators, C-BI- medium with 0.5 mg/L BAP and 0.2 mg/L IAA.

A particular primer was generated from three to seven bands ranging from 100 to 3000 bp in length. The ISSR analysis did not reveal any signs of polymorphism in any *D. forrestii* culture compared to the control shoots (Figure 5). As a consequence of the monomorphic banding patterns, all the Jaccard [25] coefficients were the same (value—1), confirming that the induced shoots were genetically homogeneous. Although only six primers were used as a tool for genetic variation studies (Table S1), it may be tentatively stated that the shoots raised on different medium compositions (including those supplemented with TRIA) maintained high uniformity, preserving high genetic similarity among the treatments.

### 2.4. Effects of Triacontanol on Activities of Antioxidant Enzymes

TRIA may affect plant antioxidant capacity by regulating the activity of some antioxidant enzymes, viz. POD, SOD and CAT [21,26]. The addition of TRIA to the MS medium was found to enhance the activity of antioxidant enzymes in *D. forrestii* shoots compared to shoots growing on C control medium (Table 1). Although all antioxidant enzymes demonstrated an increase in activity which was visible at the lowest TRIA dose, the highest activity was noted for shoots grown on MS medium with 5 µg/L TRIA: CAT 84.98 U/mg of protein, SOD 29.62 U/mg of protein, and POD 15.81 U/mg of protein (Table 1). Of these enzymes, CAT reacted most strongly to the presence of TRIA, demonstrating an eight-fold increase under optimal conditions.

The results indicate that supplementation with TRIA of the phytohormone-containing MS medium increased the activity of antioxidant enzymes compared to C-BI control treatment (MS medium with BAP and IAA) (Table 1). On the other hand, TRIA had a much weaker effect on enzyme activity when applied in the presence of growth regulators than when applied alone, while the presence of phytohormones in the medium, without TRIA, also increased the activity of all tested enzymes (C-BI vs. C). When given in combination (TRIA + BAP + IAA), CAT was the most sensitive enzyme to its presence in the medium (Table 1).

TRIA has also been found to influence enzymatic activity in other plant cultures. For example, 0.5 mL/L TRIA dosed as spray increased CAT activity *Arachis hypogaea* twenty-fold compared to the control; however, 0.5 mg/L TRIA with 0.5 mg/L BAP reduced CAT activity compared with controls containing only BAP [27].

TRIA treatment is known to increase the activity of antioxidant enzymes under normal and stress conditions. It is possible that TRIA as a signalling compound modulates the activity of the antioxidant defense system by inhibiting malondialdehyde (MDA) and H_2_O_2_ production, which could result in enhanced antioxidant levels and increased antioxidant activity [26,28,29]. TRIA is known to modulate the induction of defense-related genes responsible for water and nutrient absorption, photosynthesis, membrane stability, and enzyme activities [30]. Certain stress factors can stimulate TRIA biosynthesis and thus activate the pathways of defense reactions. However, the exogenous addition of TRIA to plants, whether by addition to nutrient medium, irrigation or foliar spraying, was found to elicit key environmental stress tolerance mechanisms as was observed in *Erythrina variegate* seedlings [31].

### 2.5. Effects of Triacontanol Phenolic Compounds Content

The stress response can also occur through non-enzymatic mechanisms. Such strategies are based on stimulating the biosynthesis of secondary metabolites that act as natural antioxidants, such as polyphenolic compounds in plants.

The levels of polyphenolic compounds in *D. forrestii* shoots grown for five weeks on MS agar media supplemented with TRIA alone and with TRIA + BAP + IAA were evaluated. The levels of the phenolic acids present in the hydromethanolic extract were determined based on their retention times and UV and MS spectra, as described previously [18]. The quantities were determined using HPLC. The total phenolic acid content was calculated as the sum of those of all analysed compounds. It was found that TRIA had a significant effect on polyphenol accumulation in *D. forrestii* culture: total phenolic acid content increased with TRIA level in the medium. The polyphenol level found in shoots grown on MS medium with 10 µg/L TRIA (12.52 mg/g DW) was 54% higher than in shoots grown on a control hormone-free medium (C) (Table 2). Supplementing the medium with phytohormones (BAP + IAA) slightly increased the total phenol content, but significantly higher polyphenol production was observed for TRIA + BAP + IAA (Table 2). The highest level of polyphenols (almost 20 mg/g DW) was achieved in shoots cultivated on medium with phytohormones and 10 µg/L TRIA, i.e., more than twice as high as in the control C-BI.

The predominant compound in all tested extracts was rosmarinic acid (RA), whose level increased with the concentration of TRIA. Rosmarinic acid is a valuable plant metabolite with wide anti-oxidant, anti-inflammatory, and anticancerogenic activities [32]. It has been found to exert hepatoprotective effects in vitro and in vivo: RA reduced liver fibrosis grade, ameliorated biochemical indicators, and downregulated TGF-β1 and CTGF expression [33]. RA also exerted a neuroprotective effect on spinal cord injury by inhibiting the TLR4/NF-κB pathway and activating Nrf2/HO-1 [34]. Khamse et al., [35] confirm that RA has strong activity against temporal lobe epilepsy. In addition, incubation with RA caused a decrease in breast cancer cell migration and induced their apoptosis [36], and a study on a rat model showed RA to offer potential as a chemopreventive agent against colon cancer [37].

The total polyphenol content in the *D. forrestii* shoots was found to be primarily influenced by changes in RA accumulation. The RA content doubled in both the medium with TRIA alone and the medium with TRIA + BAP + IAA (Table 2). TRIA appeared to have a much weaker influence on the other metabolites: a visible decrease in caffeic acid level was observed with higher TRIA concentration. This may be related to its use as a substrate in the biosynthesis of RA, whose accumulation increases significantly.

Other studies report TRIA application to result in significant increases in alkaloid production in *C. roseus* [38], the morphine level in *Papaver somniferum* [13], and artemisinin content in *Artemisia annua* [9]. However, little data has confirmed that triacontanol can influence the accumulation of phenolic compounds in plants. TRIA application has been found to increase the total phenolic level in *Zea mays* [39], and the use of a medium containing TRIA and BAP and IAA resulted in a 30% increase in RA content in *Salvia officinalis* shoots compared to controls [14].

## 3. Material and Methods

### 3.1. Plant Material and Experimental Setup

The plant material used in the experiment was *D. forrestii* shoots cultured under previously-established optimal conditions, i.e., on an MS [40] agar medium with 0.5 mg/L BAP and 0.2 mg/L IAA [18]. The nodal segments of shoots (about 0.5–1 cm in length) were used as explants.

The explants were placed on MS solid medium containing TRIA at concentrations of 2.5, 5, or 10 µg/L, and TRIA at the same concentrations, together with 0.5 mg/L BAP and 0.2 mg/L IAA. Shoots growing on MS medium without growth regulators (control C) and on medium containing 0.5 mg/L BAP and 0.2 mg/L IAA (control C-BI) were used as controls. The *D. forrestii* shoot culture was grown in a growth chamber at 26 ± 2 °C, under fluorescent lamps with a 16 h light/8 h dark photoperiod. The growth period of the culture was five weeks.

### 3.2. Measurement of Growth and Chlorophyll Content

After week five, the percentage of explants forming new buds and explants was estimated for each treatment. The number of shoots or buds per explant (proliferation ratio), as well as culture dry weight (g/tube) (DW), was recorded. The experiment was repeated three times.

To determine the chlorophyll content, the fresh biomass (0.2 g) of shoots was macerated using cool (4 °C) 80% acetone [41]. The pigment contents were established spectrophotometrically. The absorbance was tested at the following wavelengths: 664 nm for chlorophyll a and 647 nm for chlorophyll b [42]. The level of chlorophyll was expressed in mg/g FW as the sum of chlorophyll a and chlorophyll b.

### 3.3. Genetic Stability

DNA extraction from the regenerated shoots was performed following the modified CTAB protocol [43]. The plant material was weighed to reach 100 mg and then ground in liquid nitrogen. After suitable preparation, the quality of the obtained DNA was assessed by 0.7% agarose gel electrophoresis (Bio-Rad). The genomic DNA was subjected to ISSR-PCR as described by Grzegorczyk-Karolak et al. [44].

The ISSR-PCR amplification used six primers representing the UBC collection (University of British Columbia, Vancouver, Canada) (Table 1). The size of the DNA fragments was evaluated using a FastGene 100 bp DNA Marker (NIPPON Genetics Düren, Germany; length range between 100 and 3000 bp). The amplification was performed three times and the reproducible bands were scored. The genetic stability of the shoots regenerated under different medium conditions was estimated using Jaccard’s coefficient [25]: a value of 0 indicates no set similarity, while 1 indicates complete overlapping.

### 3.4. Determination of Phenolic Compound Content

Lyophilized shoots of *D. forrestii* (100 mg amounts) were extracted with 80% methanol by sonification (UD-20 ultrasonic disintegrator). The extraction is described in more detail by Weremczuk-Jeżyna et al. [18].

The plant material was subjected to quantitative HPLC analysis as described by Weremczuk-Jeżyna et al. [18]. The HPLC system consisted of a diode array detector (Waters 2998), a binary HPLC pump (Waters 2545), and an autosampler (Waters 2767). Separations were carried out on an XBridge C18 OBD column. The mobile phase (A) consisted of 0.1% trifluoroacetic acid in the water, with mobile phase B consisting of 0.1% trifluoroacetic acid in acetonitrile. The flow rate was 1.6 mL/min. UV spectra were recorded at 190–700 nm and chromatograms were acquired at 325 nm. The phenolic compounds were identified by comparing their UV spectra and retention times with those of reference compounds and/or literature data [18]. Phenolic standards such as rosmarinic acid, caffeic acid, and chlorogenic acid were purchased from Sigma-Aldrich (Darmstadt, Germany), and salvianolic acid B was purchased from Extrasynthese (Genay, France). Compounds for which pure standards were not available were quantified according to the calibration curve of similar standards.

### 3.5. Estimation of Activities of Antioxidant Enzymes

For analysis, 500 mg fresh weight of *D. forrestii* shoots was ground with a 4 mL phosphate buffer (pH = 7.5) and 0.5 mM EDTA (in 4 °C). The mixture was centrifuged (12,000 rpm for 10 min) and the supernatant was used to evaluate the activity of superoxide dismutase (SOD), catalase (CAT) and peroxidase (POD) using a UV-1800 UV/VIS Spectrophotometer (China). The CAT activity was determined at 240 nm as described by Sirivarasai et al. [45]. The POD activity was evaluated according to Hemeda and Klein [46] at a wavelength of 470 nm. SOD activity was estimated based on the ability to inhibit the reduction of nitro blue tetrazolium (NBT) (Sigma-Aldrich, Darmstadt, Germany) at 560 nm, as described by Giannopolitis and Reis [47]. The results were expressed as units per mg of protein content. Protein concentration was determined spectrophotometrically according to Bradford [48] using bovine serum albumin (Sigma-Aldrich, Darmstadt, Germany) as standard.

### 3.6. Statistical Analysis

All results were presented as means ± SE (standard error). All variables were compared using a Tukey’s HSD test. The analysis was performed using Statistica v.10 Pl for Windows (StatSoft, Krakow, Poland). *p* < 0.05 was regarded as significant.

## 4. Conclusions

In the present study, it was demonstrated that the stress-signalling mediator, triacontanol, could induce biochemical and physiological modifications in in vitro plant culture. Hence, it could be used to modulate/enhance the productivity and quality of the important plant species. However, its pattern of interaction with other hormones is ambiguous. In addition, in our study, no permanent interactions between TRIA and cytokinins were observed. However, it seems justified to conclude that the direction of TRIA-triggered action may be dose-dependent, although the type and level of response variation between species still have to be evaluated. In the given research better results were achieved, both for TRIA applied alone and along with BAP and IAA. TRIA successfully improved the multiplication ratio and growth of *D. forrestii* shoot culture at the concentrations applied in the present study. Additionally, TRIA activated the shoot defense system and increased the activity of antioxidant enzymes. It also increased the production of secondary metabolites with antioxidant properties, including rosmarinic acid, which is a compound widely used in pharmacy, cosmetology, and the food industry.

## Figures and Tables

**Table 1:** Effect of triacontanol on the antioxidant enzyme activities in *D. forrestii* shoots cultured for five weeks on MS medium containing TRIA (µg/L) or TRIA and 0.5 mg/L BAP and 0.2 mg/L IAA (TRIA-BI). C-control medium without growth regulators, C-BI- control-medium with 0.5 mg/L BAP and 0.2 mg/L IAA.

	Activity of Antioxidant Enzymes (U/mg of Protein)		
Medium with	POD	SOD	CAT
TRIA 2.5	7.81 ± 0.64 ^d^	25.11 ± 0.15 ^b^	35.17 ± 0.15 ^d^
TRIA 5	15.81 ± 0.31 ^a^	29.62 ± 0.21 ^a^	84.98 ± 0.27 ^a^
TRIA 10	7.39 ± 0.61 ^d^	23.48 ± 0.09 ^c^	71.72 ± 0.28 ^c^
C	3.21 ± 0.02 ^f^	12.10 ± 0.08 ^g^	11.75 ± 0.09 ^g^
TRIA 2.5-BI	9.54 ± 0.70 ^c^	20.05 ± 0.11 ^d^	37.15 ± 0.19 ^e^
TRIA 5-BI	11.14 ± 0.11 ^b^	16.23 ± 0.11 ^e^	78.07 ± 0.49 ^b^
TRIA 10-BI	5.07 ± 0.40 ^e^	15.78 ± 0.18 ^f^	78.49 ± 0.23 ^b^
C-BI	5.90 ± 0.09 ^e^	15.03 ± 0.12 ^f^	22.48 ± 0.18 ^f^

The values represented means ± SE of three independent experimental replicates. The means marked with various letters for the same parameter were different at *p* < 0.05 according to a Tukey’s test.

**Table 2:** The effect of TRIA on the accumulation of phenolic compounds in *D. forrestii* shoot cultured five weeks on MS medium containing triacontanol (TRIA) (µg/L) or triacontanol and 0.5 mg/L BAP and 0.2 mg/L IAA (TRIA-BI), C-medium without growth regulators.

Compound	TRIA 2.5	TRIA 5	TRIA 10	C	TRIA 2.5-BI	TRIA 5-BI	TRIA 10-BI	C-BI
chlorogenic acid	0.53 ± 0.01 ^c^	0.31 ± 0.01 ^d^	0.11 ± 0.03 ^f^	1.64 ± 0.05 ^a^	0.15 ± 0.003 ^e^	0.18 ± 0.001 ^e^	0.19 ± 0.003 ^e^	0.60 ± 0.013 ^b^
caffeic acid	0.03 ± 0.002 ^c^	0.03 ± 0.001 ^c^	0.04 ± 0.01 ^bc^	0.04 ± 0.002 ^bc^	0.10 ± 0.002 ^a^	0.09 ± 0.001 ^a^	0.09 ± 0.002 ^a^	0.05 ± 0.001 ^b^
salvianolic acid I/H and salvianolic acid E	0.45 ± 0.01 ^b^	0.28 ± 0.01 ^d^	0.28 ± 0.01 ^d^	0.67 ± 0.02 ^a^	0.36 ± 0.003 ^c^	0.41 ± 0.01 ^b^	0.44 ± 0.01 ^b^	0.45 ± 0.04 ^b^
rosmarinic acid	9.51 ± 0.22 ^d^	9.19 ± 0.23 ^d^	11.30 ± 0.02 ^b^	5.02 ± 0.01 ^f^	10.67 ± 0.21 ^c^	10.67 ± 0.19 ^c^	14.79 ± 0.31 ^a^	6.47 ± 0.01 ^e^
salvianolic acid B	0.19 ± 0.001 ^e^	0.19 ± 0.01 ^e^	0.28 ± 0.002 ^c^	0.23 ± 0.01 ^d^	0.47 ± 0.01 ^a^	0.27 ± 0.001 ^c^	0.21 ± 0.00 ^d^	0.37 ± 0.01 ^b^
methyl rosmarinate	0.28 ± 0.01 ^f^	0.39 ± 0.003 ^e^	0.48 ± 0.03 ^d^	0.54 ± 0.003 ^cd^	0.97 ± 0.04 ^a^	0.72 ± 0.03 ^b^	0.72 ± 0.01 ^b^	0.59 ± 0.1 ^c^
Total phenol content	10.96 ± 0.1 ^c^	10.39 ± 0.3 ^c^	12.52 ± 0.2 ^b^	8.14 ± 0.2 ^d^	12.72 ± 0.1 ^b^	12.35 ± 0.2 ^b^	19.44 ± 0.34 ^a^	8.53 ± 0.1 ^d^

The values represented means ± SE of three independent experimental replicates. The means marked with various letters for the same parameter were different at *p* < 0.05 according to a Tukey’s test.

## Data Availability

The data presented in this study are available from the corresponding author on request.

## Supplementary Materials

The following supporting information can be downloaded at: https://www.mdpi.com/article/10.3390/ijms232315147/s1.
