# Supplementary material for: The Effect of the Stress-Signalling Mediator Triacontanol on Biochemical and Physiological Modifications in Dracocephalum forrestii Culture
Source: Int J Mol Sci. 2022 Dec 2;23(23):15147. doi: 10.3390/ijms232315147 (PMC9735700; doi:10.3390/ijms232315147)
Supplement: Supplementary file 1 [file ijms-23-15147-s001.zip › ijms-2035959-SI.pdf]

Table S1. Primers used in ISSR-PCR analysis of *Dracocephalum forrestii* shoot treatments.

Primer Code	Primer Sequence 5′ → 3′	Annealing Temperature	Number of bands for treatment (Growth regulator combination)							
			C	C-BI	TRIA 2.5	TRIA 5	TRIA 10	TRIA 2.5-BI	TRIA 5-BI	TRIA 10-BI
ISSR-X2	CTCCTCCTCCTCRC*	45°C	3	3	3	3	3	3	3	3
UBC808	AGAGAGAGAGAGAGAGC	48°C	6	6	6	6	6	6	6	6
UBC809	AGAGAGAGAGAGAGAGG	48°C	5	5	5	5	5	5	5	5
UBC840	GAGAGAGAGAGAGAGAYT*	48°C	7	7	7	7	7	7	7	7
UBC 818	CACACACACACACACAG	48°C	5	5	5	5	5	5	5	5
UBC 834	AGAGAGAGAGAGAGAGYT*	48°C	7	7	7	7	7	7	7	7

* Y = C or T; R = A or G.
